# Is the performance at the implicit association test sensitive to feedback presentation? A Rasch-based analysis

**DOI:** 10.1007/s00426-022-01703-w

**Published:** 2022-07-08

**Authors:** Ottavia M. Epifania, Egidio Robusto, Pasquale Anselmi

**Affiliations:** Department of Philosophy, Sociology, Education, and Applied Psychology, University of Padova, Via Venezia 14, Padua, Italy

**Keywords:** Rasch model, Log-normal Model, Implicit Association Test, Feedback, Built-in correction

## Abstract

The Implicit Association Test (IAT) is commonly used for the indirect assessment of psychological constructs. While the features of the IAT that might influence the performance of the respondents have been extensively investigated, the effect of informing the respondents about the correctness of their responses (i.e., feedback presentation) has been poorly addressed so far. The study addresses this issue by presenting an across-domain (implicit prejudice and food preference) Rasch-based analysis of IAT data obtained with and without feedback presentation. Results showed that speed was influenced by the interaction between feedback presentation and associative condition, whereas accuracy was influenced by the associative condition. This result varied across-domain. Results suggested that IATs administered with feedback presentation provide more accurate information on the construct of interest.

Throughout the past two decades, the indirect investigation of socio-psychological constructs has become vastly popular in social sciences. As opposed to direct (or explicit) assessments where respondents are overtly asked to report their feelings, attitudes, and opinions regarding different topics, indirect (or implicit) assessments infer respondents’ mental states from their performance at different tasks (Greenwald & Banaji, 2017; Greenwald & Lai, 2020). Several implicit measures are available, such as the Implicit Association Test (IAT; Greenwald et al., 1998), the Go/No-go Association task (GNAT; Nosek & Banaji, 2001), the sorting paired features task (SPF; Bar-Anan et al., 2009), the Affect Misattribution Procedure (AMP; Payne et al., 2005), the Single Category IAT (SC-IAT; Karpinski & Steinman, 2006), the Brief IAT (B-IAT; Sriram & Greenwald, 2009), and the Evaluative Priming Task (EPT; Fazio et al., 1986). Among the above-mentioned measures, the IAT shows the best psychometric characteristics (Bar-Anan and Nosek, 2014). The IAT is used for the investigation of ever wider and more varied range of fields (see Epifania et al., 2021, for an extensive review on the topic), although recently its construct and criterion validities have been called into question, especially for what concerns the assessment of racial prejudice (e.g., Carlsson & Agerström, 2016; Oswald et al., 2015; Schimmack, 2021). In this light, scholars have been advised to be cautious in using the IAT for predicting real-life discriminatory behaviors. Nonetheless, once its potential limitations are called out and are cautiously taken into account, the IAT can still be considered as a useful measure for the investigation of attitudes, preferences, and stereotypes (Carlsson and Agerström, 2016). The features of the IAT procedure influencing the performance of the respondents and the most appropriate methods for scoring its data have been thoroughly investigated (Bluemke & Friese, 2006; Epifania et al., 2020; Greenwald et al., 1998,, 2003; Richetin et al., 2015). However, the effect of informing the respondents about the correctness of their responses (i.e., feedback presentation) during the administration of the IAT has been poorly addressed so far (Richetin et al., 2015). This study aims at filling this gap by presenting an across-domain Rasch analysis of IAT accuracy and time responses obtained with and without feedback presentation.

The IAT assesses the strength of automatic associations between two targets (e.g., Black people and White people in a Race IAT) and two attribute categories (i.e., Good and Bad). The measure is based on the speed and accuracy with which prototypical exemplars (appearing sequentially at the center of the computer screen) are assigned to their own category (displayed at the top corners of the screen) with two response keys. The categorization task takes place in two associative conditions, depicted in Fig. 1.

**Fig. 1:**
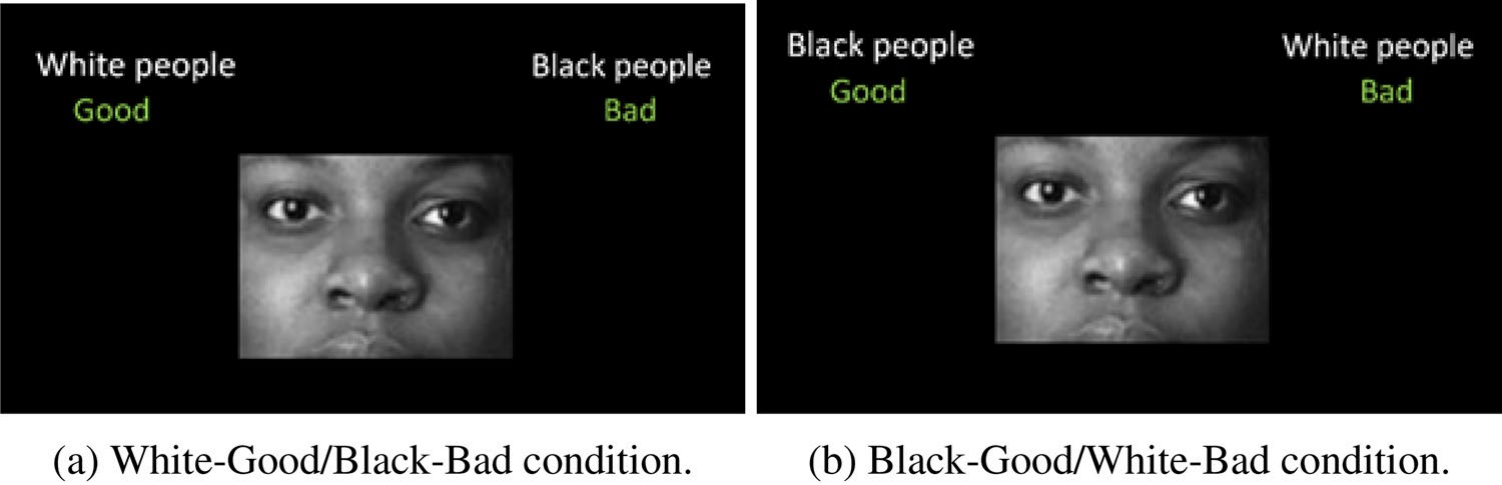
Associative conditions of a Race IAT

In one associative condition (i.e., White-Good/Black-Bad condition, Fig. 1a), the labels *White people* and *Good* share the same side of the screen. The exemplars of these categories are assigned with the same response key (e.g., E). The labels *Black people* and Bad are on the opposite side of the screen. The exemplars of these categories are assigned with another key (e.g., I). In the contrasting associative condition (i.e., Black-Good/Black Bad condition, Fig. 1b), the labels *Black people* and *Good* share the same side of the screen. The exemplars of these categories are assigned with the same response key (e.g., E). The labels *White people* and *Bad* are located on the opposite side of the screen. The exemplars of these categories are assigned with the opposite response key (e.g., I). In most applications of the IAT, feedback might be presented for each stimulus assigned with the incorrect response key. In such cases, a red X appears on the screen every time a stimulus is assigned to the wrong side of the screen. Respondents have to “correct” their response by pressing the correct response key and assigning the stimulus to the correct side of the screen to continue with the experiment. In other applications, such as those involving the Personalized IAT (P-IAT; Olson & Fazio, 2004), the incorrect responses are not followed by feedback and respondents are not required to correct their responses to continue with the experiment. Responses are expected to be faster and more accurate in the condition consistent with the automatically activated associations of the respondents. The IAT effect results from the difference in the performance of the respondents between the associative conditions. The direction and strength of the IAT effect are usually expressed by the so-called *D* scores (Greenwald et al., 2003). *D* scores are computed by dividing the difference in the average response times between associative conditions by the standard deviation of the pooled trials of both conditions. Six different *D* score algorithms are available, which differ from one another according to the treatment used for incorrect and fast responses. Incorrect responses can be replaced by either the time needed to correct them during the administration (i.e., built-in correction) or a fixed penalty (i.e., post-hoc correction). Fast responses (i.e., responses below 400 ms) can be deleted or not. According to Greenwald et al. (1998,, 2003), the performance of the respondents and the *D* score computation are not affected by feedback presentation. Therefore, all algorithms can be used interchangeably without altering the results. However, more recent evidence suggests an effect of feedback presentation on both the *D* score and the performance at the task (Ellithorpe et al., 2015; Olson & Fazio, 2004; Olson et al., 2009; Richetin et al., 2015). Specifically, the *D* score shows worse reliability and validity when the penalty used for replacing the incorrect trials is not consistent with the administration strategy (i.e., a post-hoc correction is applied on data obtained with feedback presentation) than when it is consistent (Richetin et al., 2015). Presenting feedback at each incorrect response might force the respondents to reconsider the categorization flagged as wrong and to rehearse the association required to perform correctly at the task, this increasing the accessibility of the association itself (Ellithorpe et al., 2015). Over time, the feedback presentation combined with the instruction of correcting the response might make the associations more accessible and potentially stronger, or even teach new associations to the respondents. As pointed out by Ellithorpe et al. (2015, p. 239): “If this negative feedback were to occur in such a way that it encouraged an increase in association between negative and African American, for example, it could not only hinder proper measurement of the true attitude, but could encourage a problematic and undesirable change in the participant’s attitude and the accessibility of that attitude”. As such, the automatic associations of the respondents can be confounded with the effects of the administration procedure (Ellithorpe et al., 2015; Olson & Fazio, 2004; Olson et al., 2009). The comparison between the IAT and the P-IAT (i.e., a variant of the IAT without feedback presentation) highlights a potential effect of the feedback presentation on the performance of the respondents. However, other procedural differences between the two measures (e.g., Good and Bad labels in the IAT are replaced by I like and I don’t like labels in the P-IAT) do not make possible to entirely ascribe the variations in the performance to the feedback presentation. Other evidence (Mensen et al., 2021; Szalma, 2009) further suggests that the feedback presentation either improves the performance or influences the speed-accuracy trade-off in a Go/No-go task (i.e., the sustained attention response task). Finally, since it is not always possible to ascertain whether the IAT administration included feedback presentation or not (Ellithorpe et al., 2015), understanding whether feedback presentation does affect the performance may stress the need for better reporting in IAT studies to better understand the obtained results.

Despite empirical and theoretical evidence suggest an effect of feedback presentation on the performance of the respondents at tasks similar to the IAT, this effect on the performance at the IAT has been poorly investigated. So far, the studies that have considered the feedback presentation in the IAT case either compared the performance at the IAT with the performance at another similar implicit measure (i.e., the Personalized IAT; Olson & Fazio, 2004) or did not consider feedback presentation as the main focus of the study (Ellithorpe et al., 2015). In this study, the effect of feedback presentation on the performance at the two common types of IAT is investigated in a purely exploratory fashion. Grounding on the literature concerning tasks similar to the IAT, it could be speculated that respondents might be faster when feedback is presented.

A better comprehension of the feedback effect on the performance at the IAT might be obtained by considering the interplay between the respondent’s performance and the stimulus functioning. From the respondent’s side, it would be possible to investigate if and how feedback presentation influences time and accuracy with which they perform at the task. From the stimulus side, it would be possible to investigate the variations in the time each stimulus requires for a response or the number of correct responses it obtains according to whether the feedback is presented or not. Having a detailed information at both levels might help in understanding whether the measure resulting from the IAT can be considered as a valid measure of the construct or as affected by artifacts related to the task itself. For this reason, the feedback effect on the performance at the IAT is investigated with models that are able to disentangle the unique contribution of respondents and stimuli to the observed responses, namely the Rasch model (Rasch, 1960) for accuracy responses and the log-normal model (van der Linden, 2006) for log-time responses. More detailed information on Rasch and log-normal models are given in the Method section. Rasch and log-normal parametrizations of the data are obtained with liner mixed-effect models (LMMs) to account for the fully-crossed structure of the IAT and its related sources of dependency and variability (Epifania et al., 2021; Westfall et al., 2014; Wolsiefer et al., 2017). As such, more reliable estimates can be obtained and results can be generalized at both respondent and stimulus levels simultaneously (Judd et al., 2012,, 2017; Raaijmakers et al., 1999; Raaijmakers, 2003). This approach has already proved its suitability for the analysis of the IAT and of other implicit measures (Epifania et al., 2021; Wolsiefer et al., 2017). Additionally, LMMs allow for addressing the sources of variability in within-subjects experimental designs, which helps in disentangling the variability due to experimental effects from the measure-specific one. Therefore, the importance of the experimental effects can be better understood and not confused with random noise in the data.

## Method

### Materials and procedure

A Race IAT for the assessment of racial prejudice and a Chocolate IAT for the assessment of chocolate preference were used in a $2 \times 2$ within-subjects design. These IATs are representative of two common fields of application of the IAT, namely racial prejudice and food preference (e.g., Epifania et al., 2021; Greenwald et al., 2009). It is likely that the IAT for the assessment of racial prejudice involves social desirability that might affect the responses to explicit measures, while the second does not (e.g., Greenwald et al., 2009; Richetin et al., 2015). The within-subjects factors were the type of IAT (Race IAT vs. Chocolate IAT) and the administration strategy (feedback vs. no feedback), resulting in 4 IATs (i.e., one Race IAT with feedback presentation and one without, one Chocolate IAT with feedback presentation and one without). The order of presentation of the IATs was counterbalanced across respondents, as well as the presentation of the questionnaire for the direct investigation of preferences and attitudes (either at the beginning or at the end of the experiment). Data were collected in a laboratory setting with Inquisit 3.0. In all IATs, sixteen attributes were used to represent the evaluative dimensions *Good* (i.e., *good*, *peace*, *laughter*, *glory*, *pleasure*, *joy*, *love*, *happy*) and *Bad* (i.e., *hate*, *evil*, *bad*, *terrible*, *horrible*, *harmful*, *disaster*, *failure*). In the Race IAT, twelve images were used to represent the targets *Black people* (6 images) and *White people* (6 images), same as those in Nosek et al. (2002). In the Chocolate IAT, twelve images were used to represent the targets *Dark* (6 images) and *Milk* (6 images), same as those in Epifania et al. (2020). Each IAT was composed of two critical conditions of 60 trials each, resulting in 120 trials in each IAT. As such, each respondent had 240 observations in the Chocolate IAT and 240 observations in the Race IAT, across administration strategies. The associative conditions of the Race IAT were the White-Good/Black-Bad condition (WGBB) and the Black-Good/White-Bad condition (BGWB). The associative conditions of the Chocolate IAT were the Dark-Good/Milk-Bad condition (DGMB) and the Milk-Good/Dark Bad condition (MGDB). Respondents were asked about their attitudes toward White and Black people (6-point Likert scale from 1—I strongly prefer *White people over Black people* to 6—*I strongly prefer Black people over White people*), and about their political orientation (from 0—*Liberal* to 6—*Conservative*). Chocolate preferences were investigated with two items (*How much do you like milk chocolate?* and *How much do you like dark chocolate?*) evaluated on a 6-point Likert-type scale (from 0—*Not at all* to 5—*Very much*). At the end of the experiment, participants were offered with free bars of chocolate. They were free to choose either dark or milk chocolate bars, both, none of them. The experimenter registered the choice after they left the laboratory.

### Participants

Having collected 240 observations from 142 respondents ($F = 64.08$%, Mean age $= 22.50 \pm 3.34$ years), a grand total of 34,080 observations were  available in each IAT type (i.e., Chocolate IAT vs. Race IAT) across administration strategies (i.e., Feedback vs. No Feedback). In this experiment, a high within-participant variability is expected due to the multiple observations on the same respondent. As such, power depends on both the number of trials presented to each respondent (i.e., the number of observations collected on each respondent) and the sample size (Baker et al., 2021; Westfall et al., 2014). To detect a mean difference of 0.50 with a probability of 70% and considering 240 observations on each respondent, 115 respondents suffice (power computed with the online application by Baker et al., 2021, which is available at https://shiny.york.ac.uk/powercontours/). Besides the 70% probability of successfully identifying a mean difference of 0.50, the number of observations available in the present study (i.e., 34,080) should ensure enough variability for the models to converge (Barr et al., 2013).

The respondents were informed about the confidentiality of the data and were asked for their consent to take part in the study. Most of the participants were students (93.66%). Participants did not receive any incentives for their participation to the experiment.

### Data cleaning and D score

IAT *D* scores were computed with the implicitMeasures package Epifania et al. (2020b) in R (R Core Team, 2018). A user-friendly online application for the *D* score computation is available at https://fisppa.psy.unipd.it/DscoreApp/ (i.e., DScoreApp; Epifania et al., 2020). *D1* algorithm (i.e., trials slower than 10,000 ms are discarded, incorrect trials are replaced with built-in correction, no lower tail treatment) was used to score the IAT with feedback presentation. *D3* algorithm (i.e., trials slower than 10,000 ms are discarded, incorrect trials are replaced with the average response time of the block inflated by two times the standard deviation of the block, no lower tail treatment) and *D4* algorithm (i.e., trials slower than 10,000 ms are discarded, incorrect trials are replaced with the average response time of the block inflated by 600 ms, no lower tail treatment) were used to score the IAT without feedback presentation. Positive *D* scores indicated either a preference for White people over Black people (Race IAT) or a preference for dark chocolate over milk chocolate (Chocolate IAT).

## Rasch model, log-normal model, and linear mixed-effects models

According to the Rasch model (Rasch, 1960), the characteristics of the respondent (i.e., ability) and those of the stimulus (i.e., difficulty) lie on the same latent trait (i.e., the construct of interest). As such, it is possible to consider the probability of a correct response as a function of the distance on the latent trait between the respondent’s ability and the stimulus difficulty:

1 $$\begin{aligned} P(x_{ps} = 1|\theta _p, b_s) = \frac{\exp {(\theta _p - b_s)}}{1 + \exp (\theta _p - b_s)}, \end{aligned}$$

where $P(x_{ps} = 1)$ is the probability of respondent *p* to correctly respond to stimulus *s*, $\theta _p$ is the amount of latent trait of respondent *p* (i.e., ability parameter) and $b_s$ is the amount of latent trait required by item *s* to obtain a correct response (i.e., difficulty parameter). The higher the value of $\theta _p$, the higher the ability of respondent *p* and the higher the number of correct responses provided by *p*. The higher the value of $b_s$, the higher the difficulty of stimulus *s* and the lower the number of correct responses given to *s*. If $\theta _p > b_s$, the probability of a correct response is greater than 0.50 ($P(x_{ps} = 1|\theta _p, b_s) > 0.50$). Vice versa, if $\theta _p < b_s$, the probability of a correct response is less than 0.50 ($P(x_{ps} = 1|\theta _p, b_s) < 0.50$). The Rasch model can be equated to the inverse of the *logit* link function in generalized linear models (GLMs) for binomially distributed responses (De Boeck et al., 2011; Doran et al., 2007; Epifania et al., 2021). The relationship between respondent and stimulus characteristics switches from $\theta _p - b_s$ (Rasch model) to $\theta _p + b_s$ (GLM). As such, $b_s$ can be considered as the easiness of stimulus *s*. The higher the value of $b_s$, the higher the easiness of stimulus *s* and the higher the number of correct responses *s* receives. The parametrization of the Rasch model in terms of stimulus easiness will be used in the present article.

In the log-normal model (van der Linden, 2006), the characteristics of the respondents (i.e., speed) and those of the stimulus (i.e., time intensity) lie on the same latent trait. The observed log-time response is a function of their interplay:

2 $$\begin{aligned} t_{ps} = \delta _s - \tau _p, \end{aligned}$$

where $t_{ps}$ is the expected log-time response of respondent *p* to stimulus *s*, $\delta _s$ is the time absorbing power of stimulus *s* (i.e., time intensity parameter), and $\tau _p$ expresses the speed with which respondent *p* performs the task (i.e., speed parameter). The higher the value of $\delta _s$, the higher the amount of time spent on *s*. The higher the value of $\tau _p$, the smaller the amount of time *p* spends on the stimuli. The log-time response is expected to be faster when $\tau _p > \delta _s$ than when $\tau _p < \delta _s$. The log-normal model can be easily equated to linear models (LMs) with identity functions. The relationship between respondent and stimulus characteristics switches from $\delta _s - \tau _p$ (log normal model) to $\delta _s + \tau _p$ (linear model). The interpretation of $\tau _p$ is reversed, such that the lower the value of $\tau _p$, the higher the speed of respondent *p* (the faster the log-time responses). The parametrization of the log-normal model with the reverse interpretation of the speed parameter will be used in the present article.

(Generalized) linear mixed-effects models ((G)LMMs) are obtained by including the random effects in the linear combination of predictors. Rasch and log-normal model estimates are obtained by adding the marginal modes of each level of the random effects (i.e., best linear unbiased predictors, BLUPs) to the estimates of the fixed effects. As such, (G)LMMs allows for obtaining Rasch and log-normal parametrizations from accuracy and log-time responses, respectively, while addressing the sources of random variability in the data (De Boeck et al., 2011; Doran et al., 2007). In all models, the fixed intercept is set at 0 (i.e., none of the levels of the fixed slope is taken as the reference one). The Rasch and log-normal parametrizations and the models structure are outlined in Table 1.

**Table 1:** Overview of the model estimates and lme4 notation

Model	Respondent estimates	Stimulus estimates	lme4 notation
1	$\theta _p$ or $\tau _p$	$b_s$ or $\delta _s$	${\texttt {y }\sim \texttt {0 + condition + (1|stimuli) + (1|respondents)}}$
2*s*	$\theta _p$ or $\tau _p$	$b_{sc}$ or $\delta _{sc}$	${\texttt {y }\sim \texttt {0 + condition + (0 + condition|stimuli) + (1|respondents)}}$
2*p*	$\theta _{pc}$ or $\tau _{pc}$	$b_s$ or $\delta _s$	${\texttt {y }\sim \texttt {0 + condition + (1|stimuli) + (0 + condition|respondents)}}$
3*s*	$\theta _p$ or $\tau _p$	$b_{sc}$ or $\delta _{sc}$	${\texttt {y }\sim \texttt {0 + condition + administration + (0 + condition|stimuli) + (1|respondents)}}$
3*p*	$\theta _{pc}$ or $\tau _{pc}$	$b_s$ or $\delta _s$	${\texttt {y }\sim \texttt {0 + condition + administration + (1|stimuli) + (0 + condition|respondents)}}$
4*s*	$\theta _p$ or $\tau _p$	$b_{sc}$ or $\delta _{sc}$	${\texttt {y }\sim \texttt {0 + condition }\times \texttt {administration + (0 + condition|stimuli) + (1|respondents)}}$
4*p*	$\theta _{pc}$ or $\tau _{pc}$	$b_s$ or $\delta _s$	${\texttt {y }\sim \texttt {0 + condition }\times \texttt {administration + (1|stimuli) + (0 + condition|respondents)}}$
5*s*	$\theta _{p}$ or $\tau _{p}$	$b_{sck}$ or $\delta _{sck}$	${\texttt {y }\sim \texttt {0 + condition }\times \texttt {administration + (0+condition:administration|stimuli) + (1|respondents)}}$
5*p*	$\theta _{pck}$ or $\tau _{pck}$	$b_s$ or $\delta _s$	${\texttt {y }\sim \texttt {0 + condition }\times \texttt {administration + (1|stimuli) + (0 + condition:administration|respondents)}}$

*s*: random slopes are allowed at stimulus level, *p*: random slopes are allowed at respondent level. From Model 3, the level on which the multidimensionality is allowed depends on the best fitting model between Models 1, 2*s* and 2*p*. $p = 1, \ldots , P$, Stimulus $s = 1,\ldots , S$, Condition $c = 1,\ldots , C$, Administration $k = 1, \ldots , K$, where *P*, *S*, *C*, and *K* denote the number of respondents, stimuli, conditions, and administration procedures, respectively. $\theta$: respondent ability estimates, $\tau$: respondent speed estimates, *b*: stimulus easiness estimates, $\delta$ stimulus time intensity estimates. The dependent variable *y* can be either the accuracy responses in the GLMMs or the log-time responses in the LMMs

Model 1 is the null model. This model yields overall (i.e., across associative conditions and administration procedures) respondent estimates ($\theta _p$ or $\tau _p$) and overall stimulus estimates ($b_s$ or $\delta _s$). Model 1 should be preferred when low within-respondents and within-stimuli variabilities are observed between associative conditions and administration strategies. This suggests that neither the performance of the respondents nor the functioning of the stimuli are affected by the associative condition or by the administration strategy.

Two Models 2 are specified, one where the random slopes in associative conditions are specified at stimulus level (Model 2*s*), and one where the random slopes in associative conditions are specified at respondent level (Model 2*p*). Model 2*s* yields condition-specific stimulus estimates ($b_{sc}$ or $\delta _{sc}$) where *c* denotes the associative condition and overall respondent estimates ($\theta _p$ or $\tau _p$). It should be preferred when high within-stimuli between-conditions variability is observed. This suggests that the IAT effect mostly depends on the variations in the stimulus functioning between conditions. Model 2*p* provides condition-specific respondent estimates ($\theta _{pc}$ or $\tau _{pc}$), and overall stimulus estimates ($b_s$ or $\delta _s$). It should be preferred when high within-respondents variability is observed. This suggests that the IAT effect mostly depends on the variations in respondents’ performance between conditions. Conversely, the low variability at stimulus level suggests that the functioning of the stimuli does not vary much between conditions. Model 2*s* and Model 2*p* are compared with each other and with Model 1. The model comparison between Models 1, 2*s* and 2*p* helps in understanding whether the IAT effect is mostly due to variations in respondent’s performance or stimulus functioning, and to further investigate the variations at that level by specifying the random slopes on it.

In Model 3, the administration strategy is added as a main fixed effect. In Model 4 the interaction effect between associative condition and administration is added as fixed effect. If Models 3 or 4 result as the best fitting model, it means that the feedback presentation has an effect at the sample level. In both cases, the Rasch and log-normal parametrizations are the same as those obtained from the best fitting model between Models 2*s* and 2*p*.

In Model 5, the interaction effect between associative condition and administration strategy is added to the random slopes of either respondents (Model 5*p*) or stimuli (Model 5*s*). Model 5*p* yields condition-and administration-specific respondent estimates ($\theta _{pck}$ or $\tau _{pck}$, where *k* denotes the administration strategy) and overall stimulus parameters ($b_s$ or $\delta _s$). Model 5*p* should be preferred when high within-respondents variability is observed between conditions and administrations. This suggests that the IAT effect is mostly due to the variations in respondents’ performance between conditions and it is further influenced by the feedback presentation. The differences between condition-specific respondent estimates (across administration strategy) inform about the bias due to the associative condition on the performance of the respondents. The differences between administration-specific respondent estimates (across associative conditions) inform about the bias on the performance ascribable to the administration strategy. Model 5*s* yields condition- and administration-specific stimulus estimates ($b_{sck}$ or $\delta _{sck}$), and overall respondent estimates ($\theta _p$ or $\tau _p$). This model should be preferred when high within-stimuli variability is observed between-conditions and administrations. This suggests that the IAT effect is mostly due to variations in stimulus functioning and that it is further influenced by the administration strategy. The differences between condition-specific stimulus estimates (across administration strategy) inform about the bias on the functioning of the stimuli due to the associative condition. The differences between the administration-specific stimulus estimates (across associative conditions) inform about the change in the functioning of the stimuli according to the administration strategy.

Models were fitted in R (R Core Team, 2018) with the lme4 package (Bates et al., 2015, bobyqa optimizer). From now on, the data and IATs including feedback presentation will be referred to as “Feedback data” and “Feedback IAT” whereas those not including feedback presentation will be referred to as “No Feedback data” and “No Feedback IAT”. Models applied to IAT accuracy responses are identified by a capital A, while those applied to IAT log-time responses are identified by a capital T.

## Results

Model comparison is based on Akaike’s Information Criterion (Akaike, 1974), Bayesian Information Criterion (Schwarz, 1978), deviance, and Log-Likelihood. Lower values of these indexes indicate better fit of the model.

### Accuracy models

Table 2 reports the results of accuracy models. In the Race IAT, the multidimensionality was allowed at the stimulus level because Model A2*p* produced aberrant estimates and Model A2*s* performed better than Model A1. In the Chocolate IAT, Model A2*p* performed better than Models A1 and A2*s*, hence the multidimensionality was allowed at the respondent level.

**Table 2:** Accuracy models

Race IAT						
	Model A1	Model A2*p*	Model A2*s*	Model A3*s*	Model A4*s*	Model A5*s*
BGWB	2.71$$^{***}$$	Aberrant	2.71$$^{***}$$	2.65$$^{***}$$	2.68$$^{***}$$	Singular
	(0.06)	estimates	(0.07)	(0.07)	(0.08)	fit
WGBB	3.33$$^{***}$$		3.37$$^{***}$$	3.31$$^{***}$$	3.27$$^{***}$$	
	(0.07)		(0.09)	(0.09)	(0.10)	
No feedback				0.12$$^{**}$$	0.07	
				(0.05)	(0.06)	
WGBB $\times$ No Feedback					0.12	
					(0.10)	
Observations	34,080		34,080	34,080	34,080	
AIC	14307.62		14290.08	14285.9	14286.37	
BIC	14341.37		14340.7	14344.96	14353.86	
Deviance	14,299.63		14,278.08	14,271.90	14,270.37	
Log-Likelihood	−7,149.812		−7,139.041	−7,135.951	−7,135.184	

Chocolate IAT						
	Model A1	Model A2*p*	Model A2*s*	Model A3*p*	Model A4*p*	Model A5*p*
DGMB	2.72$$^{***}$$	2.72$$^{***}$$	2.72$$^{***}$$	2.69$$^{***}$$	2.69$$^{***}$$	Singular
	(0.07)	(0.07)	(0.07)	(0.08)	(0.08)	Fit
MGDB	3.38$$^{***}$$	3.41$$^{***}$$	3.39$$^{***}$$	3.38$$^{***}$$	3.37$$^{***}$$	
	(0.08)	(0.08)	(0.08)	(0.09)	(0.09)	
No feedback				0.06	0.06	
				(0.05)	(0.06)	
MGDB $\times$ No Feedback					0.02	
					(0.10)	
Observations	34,080	34,080	34,080	34,080	34,080	
AIC	14,349.98	14,347.37	14349.59	14,347.65	14,349.62	
BIC	14,383.73	14,397.99	14,400.21	14,406.71	14,417.11	
Deviance	14,342.98	14,335.37	14,337.58	14,333.651	14333.62	
Log-Likelihood	−7,170.99	−7,167.69	−7,168.794	−7,166.83	−7,166.81	

*BGWB:* Black-Good/White-Bad condition, *WGBB:* White-Good/Black-Bad condition, *DGMB:* Dark-Good/Milk-Bad condition, *MGDB:* Milk-Good/Dark-Bad condition, *No feedback*: Administration strategy without feedback presentation. The estimates are the *log-odds* of the probability of observing a correct response, standard errors are reported in parentheses

$${***}p< 0.01$$

Although Model A2*s* showed a lower BIC in the Race IAT, AIC, deviance, and log-likelihood suggested Model A3*s* as the best fitting model. Thus, Model A3*s* was chosen, providing condition-specific easiness estimates $b_{\text {BGWB}}$ and $b_{\text {WGBB}}$ and overall ability estimates $\theta _p$ of the Rasch model. In the Chocolate IAT, Model A2*p* performed better than Models A1 and A2*s*. Thus, random slopes were specified at respondent level. Model A2*p* was the best fitting model. This model provided condition-specific ability estimates ($\theta _{\text {DGMB}}$ and $\theta _{\text {MGDB}}$), and overall easiness estimates $b_s$ of the Rasch model. In the Race IAT, the IAT effect could be mostly ascribed to variations in stimulus functioning between conditions, while in the Chocolate IAT it could be mostly ascribed to the variations in the respondents’ performance between conditions.

Stimuli of the Race IAT tended to be easier in WGBB condition than in BGWB condition ($M_{\text {WGBB} }=\ 3.35\ \pm 0.25$ and $M_{\text {BGWB}}=2.71\pm 0.12$, $t\left( 39.87\right) =12.43$, $p<0.001$, 95% CI $\left[ 0.54;0.75\right]$, $d\ =\ 3.26)$. In the Chocolate IAT, respondents showed higher ability in MGDB condition than in DGMB condition ($M_{\text {MGDB}}\ =\ 3.38\ \pm 0.50$, $M_{\text {DGDB}}=3.69\pm 0.60$, $t\left( 280.93\right) =9.87$, $p<0.001$, 95% CI $\left[ 0.55;0.82\right]$, $d\ =\ 0.53$). At sample level (i.e., fixed effects), higher percentages of correct responses were observed in the associative condition where the stimuli were easier (i.e., WGBB condition of the Race IAT) and where the respondents showed higher ability (i.e., MGDB condition of the Chocolate IAT). In the Race IAT, the No Feedback administration strategy fostered the probability of correct response at sample level, while no effect of the administration strategy was found in the Chocolate IAT. Neither the functioning of the stimuli nor the performance of the respondents were affected by the administration strategy in both Chocolate and Race IATs.

The difference between condition-specific stimulus estimates can be considered as an accuracy-based measure of the IAT effect on the stimulus functioning. It can be interpreted as the contribution of each stimulus to the IAT effect. The difference was computed between $b_{\text {WGBB}}$ and $b_{\text {BGWB}}$ estimates (i.e., higher values denote stimuli easier in the WGBB condition than in the BGWB condition). Linear models were specified to investigate the effect of the stimulus categories on the difference between condition-specific easiness estimates (Race IAT) and on the overall easiness estimates (Chocolate IAT). In both cases, significant effects of stimulus categories were found (Race IAT: $F\left( 4;24\right) =60.27$, $p<0.001$, *Adjusted R*$^2\ =\ 0.89$, Chocolate IAT: $F\left( 4;24\right) =4.34$, $p<0.001$, *Adjusted R*$^2=0.32$). In the Race IAT, *Good* and *White people* exemplars contributed the most to the IAT effect ($B_{\text {Good}} = 0.89$, $SE = 0.08$, $t(24) = 11.06$, $p <0 .001$ and $B_{\text {White}} = 0.60$, $SE = 0.09$, $t(24) = 6.34$, $p < .001$), while *Black people* and *Bad exemplars* gave a lower contribution ($B_{\text {Black}} = 0.64$, $SE = 0.08$, $t(24) = 6.83$, $p <0 .001$ and $B_{\text {Bad}} = 0.46$, $SE = 0.08$, $t(24) = 5.66$, $p < 0.001$). In the Chocolate IAT, *Milk* exemplars were the most difficult ones ($B = -0.19$, $SE = 0.05$, $t(24) = -3.44$, $p = 0.001$). Exemplars of other categories showed an average level of easiness ($B_{\text {Dark}} = -0.06$, $SE = 0.05$, $t(24) = -1.04$, $p = 0.30$, $B_{\text {Bad}} = 0.06$, $SE = 0.05$, $t(24) = 1.28$, $p = 0.21$, $B_{\text {Good}} = 0.08$, $SE = 0.05$, $t(24) = 1.67$, $p = 0.11$).

### Log-time models

The results of the log-time models are reported in Table 3. In the Race IAT, Model T2*p* performed better than Models T1 and T2*s*. In the Chocolate IAT, Model T2*s* resulted in aberrant estimates, and Model T2*p* performed better than Model T1. Consequently, random slopes were specified at respondent level in both IATs. Model T5*p* was the best fitting model in both IATs. This model provided condition- and administration-specific respondent speed estimates (Race IAT: $\tau _{\text {BGWB}}^F$, $\tau _{\text {WGBB}}^F$, $\tau _{BGWB}^{NF}$, $\tau _{\text {WGBB}}^{NF}$, Chocolate IAT: $\tau _{\text {DGMB}}^F$, $\tau _{\text {MGDB}}^F$, $\tau _{\text {DGMB}}^{NF}$, $\tau _{\text {MGDB}}^{NF}$, where superscript *F* indicates estimates from Feedback data and superscript *NF* indicates estimates from No Feedback data) and overall stimulus time intensity estimates ($\delta _s$ for each IAT) of the log-normal model.

**Table 3:** Log-time models

Race IAT						
	Model T1	Model T2*p*	Model T2*s*	Model T3*p*	Model T4*p*	Model T5*p*
BGWB	-0.26$$^{***}$$	-0.26$$^{***}$$	-0.263$$^{***}$$	-0.29$$^{***}$$	$$-0.30^{***}$$	-0.30$$^{***}$$
	(0.01)	(0.01)	(0.01)	(0.01)	(0.01)	(0.02)
WGBB	-0.43$$^{***}$$	-0.43$$^{***}$$	-0.430$$^{***}$$	-0.46$$^{***}$$	$$-0.46^{***}$$	-0.455$$^{***}$$
	(0.01)	(0.01)	(0.013)	(0.01)	(0.01)	(0.01)
No feedback				0.06$$^{***}$$	0.07$$^{***}$$	0.07$$^{***}$$
				(0.01)	(0.01)	(0.01)
WGBB $$\times$$ No feedback					-0.02$$^{**}$$	-0.02
					(0.01)	(0.01)
Observations	34,080	34,080	34,080	34,080	34,080	34,080
AIC	25,675.82	25,212.04	25,677.62	24,960.70	24,957.44	24,274.83
BIC	25,718.00	25,271.09	25,736.68	25,028.19	25,033.37	24,409.81
Log-likelihood	−12,832.91	−12,599.02	−12,831.81	−12,472.35	−12,469.72	−12,121.42
Deviance	25,666	25,198	25,198	25,663.6	24,939	24,243
Chocolate IAT						
DGMB	-0.28$$^{***}$$	-0.28$$^{***}$$	Aberrant	-0.30$$^{***}$$	-0.31$$^{***}$$	-0.31$$^{***}$$
	(0.02)	(0.02)	estimates	(0.02)	(0.02)	(0.02)
MGDB	-0.47$$^{***}$$	-0.47$$^{***}$$		-0.50$$^{***}$$	-0.49$$^{***}$$	-0.49$$^{***}$$
	(0.02)	(0.02)		(0.02)	(0.02)	(0.02)
No feedback				0.05$$^{***}$$	0.06$$^{***}$$	0.06$$^{***}$$
				(0.01)	(0.01)	(0.01)
MGDB $$\times$$ No feedback					-0.02$$^{***}$$	-0.02$$^{*}$$
					(0.01)	(0.01)
Observations	34,080	34,080		34,080	34,080	34,080
AIC	26,381.70	25,211.93		25,061.62	25,054.32	24,581.76
BIC	26,423.88	25,270.98		25,129.12	25,130.25	24,716.74
Log likelihood	−13,185.85	−12,598.96		−12,522.81	−12,518.16	−12,274.88
Deviance	26,372	25,198		25,046	25,036	24,550

*DGMB* Dark-Good/Milk-Bad condition, *MGDB* Milk-Good/Dark-Bad condition, *No feedback* administration strategy without feedback presentation. The estimates are expressed in log-seconds, standard errors are reported in parentheses

$${***} p<0.01$$; $${*} p<0.10$$

Linear models were specified to investigate the effect of the stimulus categories on the time intensity estimates. A significant effect of the stimulus categories was found in both IATs (Race IAT: $F(4; 24) = 5.66, p < 0.001$, *Adjusted R*$^2 = 0.42$, Chocolate IAT: $F(4; 24) = 29.30$, $p < .001$, Adjusted R$^2 = 0.80$). In both IATs, Bad and Good exemplars required the highest amount of time for getting a response, although the Good exemplars were not significantly different from 0 in the Race IAT (Race IAT: $B_{\text {Bad}} = 0.02$, $SE = 0.01$, $t(24) = 2.45$, $p = 0.02$, and $B_{\text {Good}} = -0.01$, $SE = 0.01$, $t(24) = -0.84$, $p = 0.40$, Chocolate IAT: $B_{\text {Bad}} = 0.04$, $SE = 0.01$, $t(24) = -7.72$, $p < 0.001$, $B_{\text {Good}} = 0.02$, $SE = 0.01$, $t(24) = 2.88$, $p = 0.001$). Black people exemplars required the least amount of time to get a response ($B = -0.03$, $SE = 0.01$, $t(24)=-3.59$, $p < 0.001$), while White people exemplars were not significantly different from 0 ($B = 0.01$, $SE = 0.01$, $t(24) = 1.73$, $p = 0.10$). Dark and Milk exemplars required the least amount of time for getting a response ($B_{\text {Dark}} = -0.06$, $SE = 0.01$, $t(24) = -7.72$, $p <0 .001$, $B_{\text {Milk}} = -0.02$, $SE = 0.01$, $t(24) = -2.96$, $p = 0.001$).

### Relationship between model estimates, explicit attitudes, and typical scores

#### Race IAT

Pearson’s correlations between explicit attitudes towards Black and White people, political orientation, *D* score algorithms, and model estimates are reported in Table 4.

**Table 4:** Race IAT correlations

	1	2	3	4	5	6	7	8	9
1-Explicit attitudes									
2-Political orientation	− 0.34***								
3-*D1*	− 0.06	0.03							
4-*D3*	0.00	0.12	0.36***						
5-*D4*	-0.02	0.11	0.37***	0.99***					
6-$\theta _p$	0.06	− 0.09	0.01	− 0.05	− 0.02				
7-$\tau _{\text {BGWB}}^F$	0.04	− 0.04	0.32***	0.05	0.06	0.40***			
8-$\tau _{\text {BGWB}}^{NF}$	0.06	0.01	0.09	0.35***	0.37***	0.28***	0.66***		
9-$\tau _{\text {WGBB}}^F$	0.06	− 0.07	− 0.30***	− 0.24**	− 0.23**	0.41***	0.77***	0.59***	
10-$\tau _{\text {WGBB}}^{NF}$	0.09	− 0.08	− 0.23**	− 0.23**	− 0.22**	0.35***	0.65***	0.77***	0.85***

*D1:* *D* score using built-in correction, *D3:* *D* score using 2sd post-hoc error penalty, *D4*: *D* score using 600 ms error penalty, $\theta$: Ability estimates, $\tau$: speed estimates, *BGWB:* Black-Good/White-Bad condition, *WGBB:* White-Good/Black-Bad, *F:* Feedback data, *NF:* No feedback data

$${***} p <0.001$$, $${**} p < 0.05$$

Political orientation correlated with attitudes towards Black people, such that the more the individuals reported right-wing orientation, the more they explicitly preferred White people over Black people. Neither political orientation nor explicit attitudes correlated with any of the implicit measures of attitudes. Ability positively correlated with feedback and no feedback speed estimates (i.e., the higher the ability, the lower the speed). In the Feedback data, ability correlated almost identically with both condition-specific speed estimates ($z = -0.30$, $p = 0.77$), this suggesting a similar speed-accuracy trade-off between conditions. Ability showed similar correlations with the condition-specific estimates of the no feedback data ($z = -1.25$, $p = 0.21$). Ability did not correlate with any of the *D* scores. The strong correlation between *D3* and *D4* suggested that they could be used interchangeably (i.e., the post-hoc correction does not affect the final score much). On the other hand, the weak correlation between *D1* and other *D* scores suggested that the penalization of incorrect trials affect the resulting scores.

#### Chocolate IAT

Pearson’s correlations between explicit chocolate evaluations, *D* scores, and model estimates are reported in Table 5.

**Table 5:** Chocolate IAT correlations

	1	2	3	4	5	6	7	8	9	10
1-Dark explicit										
2-Milk explicit	− 0.26**									
3-*D1*	0.35***	− 0.41***								
4-*D3*	0.42***	− 0.30***	0.63***							
5-*D4*	0.42***	− 0.31***	0.62***	1.00***						
6-$\theta _{\text {DGMB}}$	0.02	− 0.04	0.16	0.19*	0.16					
7-$\theta _{\text {MGDB}}$	− 0.07	0.03	0.03	0.06	0.02	0.96***				
8-$\tau _{\text {DGMB}}^F$	− 0.28***	0.29***	− 0.49***	− 0.24**	− 0.26**	0.15	0.25**			
9-$\tau _{\text {DGMB}}^{NF}$	− 0.34***	0.29***	− 0.37***	− 0.47***	− 0.51***	0.10	0.21*	0.78***		
10-$\tau _{\text {MGDB}}^F$	0.01	− 0.03	0.28***	0.32***	0.29***	0.32***	0.34***	0.64***	0.53***	
11-$\tau _{\text {MGDB}}^{NF}$	0.02	− 0.03	0.26**	0.39***	0.36***	0.27**	0.28***	0.58***	0.56***	0.94***

*D1:* *D* score using built-in correction, *D3*: *D* score using 2sd *post-hoc* error penalty, *D4:* *D* score using 600 ms error penalty, $\theta$: ability estimates, $\tau$: speed estimates, *DGMB:* Dark-Good/Milk-Bad condition, *MGDB:* Milk-Good/Dark-Bad, *F:* Feedback data, *NF:* No feedback data

$${***} p <.001$$, $${**} p <0 .05$$

The correlations between explicit chocolate evaluations and *D* scores were consistent with the direction of *D* score computation, as well as the correlations between speed estimates and *D* scores. The explicit chocolate evaluations correlated only with the speed in DGMB condition. *D* scores correlated only with speed in DGMB condition. Moreover, *D1* showed stronger correlation with speed in DGMB condition of Feedback data than of No Feedback data ($z = -2.41$, $p =0.02$). Similarly, *D3* and *D4* showed stronger correlations with speed in DGMB of No Feedback data than with speed in DGMB condition of Feedback data (*D3*: $z = 4.57$, $p <0 .001$, *D4*: $z = 4.86$, $p <0.001$)). Ability in DGMB condition did not correlate with any of the speed estimates of Feedback data. Ability in MGDB condition positively correlated with condition-specific speed in both Feedback and No Feedback data.

### Prediction of the behavioral outcome

*D* scores and model estimates of the Chocolate IAT data were used to predict the observed chocolate choice. Speed differentials were obtained by taking the difference between condition-specific speed estimates (i.e., positive scores indicate higher speed in the DGMB condition than in MGDB condition). Speed differentials can be considered as time-based measures of the IAT effect on respondents’ performance. The predictive abilities of differential measures (i.e., *D* scores and speed differentials), of their single components, and of ability estimates (i.e., $M_{\text {DGMB}}$ and $M_{\text {MGDB}}$ of the *D* scores, $\theta _{\text {DGMB}}$ $\tau _{\text {DGMB}}$, $\tau _{\text {MGDB}}$ of the speed differential, $\theta _{\text {MGDB}}$ and $\theta _{\text {DGMB}}$) were investigated. The condition-specific average response times were computed on the inflated latencies, according to the corresponding *D* score algorithm (i.e., built-in, *2sd*, 600 *ms*).

Respondents who chose both chocolate bars ($n = 5$) were excluded from the analysis. Since it was not possible to ascertain whether respondents who did not take any chocolate ($n = 41$) did so because of low levels of the latent trait (i.e., they do not like chocolate) or because of other situational factors (e.g., satiety, dieting), they were excluded from the analysis as well. Logistic regressions for predicting the dichotomous choice between dark chocolate and milk chocolate were run on the remaining sample ($n = 96$). Among the remaining 96 respondents, the 31.39% ($n = 43$) chose dark chocolate (dark chocolate choice, DCC), and the 38.69% ($n = 53$) chose milk chocolate (milk chocolate choice, MCC). Events per variable (EPV, i.e., ratio between the number of the smallest category of the dichotomous outcome and the number of regression coefficients excluding the intercept) have been considered for determining whether the sample size was adequate for running the logistic regressions on the remaining sample (e.g., Harrell et al., 1984). Ten EPV is a generally adopted minimal guideline for determining the sample size needed to perform binary logistic models (e.g., Moons et al., 2015). Given that the maximum number of predictors in the model excluding the intercept would be four (i.e., the condition-specific ability estimates and the condition-specific speed estimates or average response times) and the smallest category (MCC) is composed of 43 observations, $\text {EPV} = 43/4 = 10.75$. As such, the sample size should be adequate for running the logistic regression models and obtaining interpretable results.

All starting models included the predictors of interest and the condition-specific ability estimates. Relevant predictors were chosen with forward selection. General accuracy (i.e., ratio between model’s correctly identified choices and total number of choices), DCC accuracy (i.e., ratio between model’s correctly identified DCCs and observed DCCs), and MCC accuracy (i.e., ratio between model’s correctly identified MCCs and observed MCCs) were computed on the models resulting after forward selection (Table 6).

**Table 6:** Choice prediction: models resulting after forward selection

Model	Predictors	*B*	SE	Nagelkerke R$^2$	General	DCC	MCC
Null Model							
0	Intercept	0.21	0.21	0	0.45	1.00	0.00
Differential measures							
1	Intercept	− 0.81*	0.36	0.21	0.67	0.53	0.77
	D1	− 2.25***	0.63				
2	Intercept	− 0.55	0.32	0.15	0.68	0.58	0.75
	D3	− 1.61***	0.51				
3	Intercept	− 0.55	0.32	0.16	0.68	0.58	0.75
	D4	− 1.72***	0.53				
4	Intercept	− 1.09*	0.40	0.26	0.68	0.63	0.72
	$\tau _{\text {MGDB}}^F - \tau _{\text {DGMB}}^F$	− 7.75***	2.05				
5	Intercept	− 0.78*	0.35	0.19	0.71	0.60	0.79
	$\tau _{\text {MGDB}}^{NF} - \tau _{\text {DGMB}}^{NF}$	− 5.57***	1.59				
Single measures							
6	Intercept	− 2.90	1.59	0.31	0.67	0.63	0.70
	$M_{\text {DGMB}}^F$	0.01***	0.01				
	$M_{\text {MGDB}}^F$	0.01	0.01				
7	Intercept	− 3.95***	1.24	0.18	0.66	0.53	0.75
	$M_{\text {DGMB}}^{NF}$ (2sd)	0.01***	0.01				
8	Intercept	− 4.13***	1.28	0.19	0.64	0.53	0.72
	$M_{\text {DGMB}}^{NF}$ (600 ms)	0.01***	0.01				
9	Intercept	0.63	0.89	0.31	0.74	0.67	0.79
	$\tau _{\text {DGMB}}^F$	8.72***	2.19				
	$\tau _{\text {MGDB}}^{F}$	− 4.97*	2.40				
10	Intercept	0.92	0.76	0.27	0.70	0.60	0.77
	$\tau _{\text {DGMB}}^{NF}$	7.19***	1.85				
	$\tau _{\text {MGDB}}^{NF}$	− 2.98	1.90				

*D1* *D* score with built-in correction, *D3* *D* score with 2sd penalty, *D4* *D* score with 600 ms penalty, *DGMB* Dark/Good-Milk/Bad condition, *MGDB* Milk/Good-Dark/Bad condition, *F* feedback data, *NF* no feedback data, $\theta$ ability estimates, $\tau$ speed estimates

$${***}$$: $$p < 0.001$$, $${**}$$: $$p < 0.01$$, $${*}$$: $$p < 0.05$$

The model including the condition-specific speed estimates (Model 9) and that including the condition-specific average response time from the Feedback data (Model 6) resulted in the highest proportion of explained variance. Additionally, Model 9 resulted in the highest General accuracy of prediction, immediately followed by the model including the speed differential from No Feedback data (Model 5) and that including condition-specific speed estimates from No Feedback data (Model 10).

Among the *D* scores, *D1* resulted in the highest *R*^2^. All *D* scores resulted in approximately the same accuracies of prediction.

## Discussion and conclusions

This study investigated whether feedback presentation influences respondents’ speed and accuracy at the IAT. The results suggested that speed is affected by the interaction between associative condition and feedback presentation, while accuracy is affected only by the associative condition. However, this result varied across types of IAT.

In the Chocolate IAT, the IAT effect on the accuracy responses was mostly due to the variations in respondents’ performance. The pattern of correlations between ability estimates and explicit measures suggested a better accuracy in the condition where the preferred chocolate was associated with positive attributes than when it was associated with negative ones. In the Race IAT, the IAT effect on accuracy responses was mostly due to the variations in the functioning of the stimuli between associative conditions. Specifically, the variations between conditions of *Good* and *White people* exemplars gave the higher contribution to the IAT effect, with these exemplars being easier in the White-Good/Black-Bad condition. This result is consistent with the positive primacy effect highlighted in previous studies (e.g., Anselmi et al., 2011, 2013), according to which the IAT effect is mostly due to the associations between the positive evaluative dimension and the target representing the ingroup members. In this sense, the IAT effect should be interpreted more as an expression of ingroup favoritism rather than as an expression of outgroup derogation.

While the implicit assessments obtained from the Chocolate IAT correlated with their respective explicit assessments, those of the Race IAT did not correlate with either political orientation or attitudes towards White and Black people, irrespective of the administration strategy. The lack of correlation between explicit measures and IAT measures of racial prejudice might be due to different reasons, such as the poor construct validity of the IAT Schimmack (2021), the use of single items for the explicit assessment, and/or the fact that implicit and explicit measures of the same construct tend not to correlate when the construct under investigation is potentially prone to social desirability (Greenwald et al., 2009).

In both IATs, speed was affected by the joint effect of associative condition and feedback presentation. Respondents tended to be faster when feedback was presented than when it was not. The “speeding” effect of the feedback was more evident in the condition consistent with the automatically activated association of the respondents. This potentially suggests that respondents might adopt different speed-accuracy trade-offs according to the feedback presentation or lack of thereof, consistent with previous findings on tasks similar to the IAT (e.g., Mensen et al., 2021; Szalma, 2009). Feedback presentation makes the respondents aware of the unlikelihood of the occurrence of incorrect responses. As such, when feedback is presented a speed-accuracy trade-off that favors speed over accuracy might be chosen, this potentially leading to more confident time performances and faster response times. Conversely, the absence of feedback presentation might leave the respondents in a state of uncertainty about their accuracy, this potentially leading to more conservative speed performances and slower response times. The pattern of correlations between condition-specific ability and administration- condition-specific speed estimates in the Chocolate IAT corroborates these speculations. Specifically, in the IAT with feedback presentation higher levels of ability were also associated with higher speed (faster responses), while they were associated with lower speed (slower responses) in the IAT without feedback.

The measures obtained from Feedback data best predicted the chocolate choice. This result was more evident in the models using the condition-specific estimates than in those using differential measures. Among differential measures, the speed differential obtained from No Feedback and Feedback data best predicted both dark and milk chocolate choices. Considering the predicting performance of the *D* scores and of their single components, the ones computed with the built-in correction (hence obtained from Feedback data) best predicted the choice.

While the *D* score flattens the differences due to the administration strategies, the strong correlation between *D* scores using post-hoc corrections combined with their low correlations with *D* scores using built-in correction suggest that error replacement strategies can affect the results. As such, the specific administration strategy should always be reported in the method section to allow for a better interpretation of the results.

The use of single items to assess the construct validity of both IATs is the major limitation of the study. This was done to avoid an excessive burden on the respondents since they were already presented with four IATs. However, as pointed out in the literature (e.g., Nunnally & Bernstein, 1994), single-item assessments are more prone to measurement error and provide lower content validity than multiple-item assessments.

In conclusion, feedback presentation does influence the time performance at the IAT, regardless of the type of IAT. In contrast with what highlighted by other authors (see, e.g., Ellithorpe et al., 2015; Olson & Fazio, 2004; Olson et al., 2009), the results of this study suggest that feedback presentation during the IAT administration might provide a more valid measure of the construct because it might keep the respondents constantly engaged in the task (Szalma, 2009). Additionally, by being aware of the unlikelihood of incorrect responses, respondents seem to spend less time thinking about the correct response and to provide faster responses consistent with the automatic nature of the IAT assessment. The IATs including feedback presentation should be preferred over the ones without.

## Data Availability

Data and R script are available in the Open Science Framework at: https://osf.io/y2qak/.
